# Optical mapping of the *Mycobacterium avium *subspecies *paratuberculosis *genome

**DOI:** 10.1186/1471-2164-10-25

**Published:** 2009-01-15

**Authors:** Chia-wei Wu, Timothy M Schramm, Shiguo Zhou, David C Schwartz, Adel M Talaat

**Affiliations:** 1 The Laboratory of Bacterial Genomics, Department of Pathobiological Sciences, University of Wisconsin-Madison, WI, USA; 2 Laboratory for Molecular and Computational Genomics, Department of Chemistry, Laboratory of Genetics, University of Wisconsin-Madison, WI, USA

## Abstract

**Background:**

Infection of cattle with *Mycobacterium avium *subspecies *paratuberculosis *(*M. ap*) causes severe economic losses to the dairy industry in the USA and worldwide. In an effort to better examine diversity among *M. ap *strains, we used optical mapping to profile genomic variations between strains of *M. ap *K-10 (sequenced strain) and *M. ap *ATCC 19698 (type strain).

**Results:**

The assembled physical restriction map of *M. ap *ATCC 19698 showed a genome size of 4,839 kb compared to the sequenced K-10 genome of 4,830 kb. Interestingly, alignment of the optical map of the *M. ap *ATCC 19698 genome to the complete *M. ap *K-10 genome sequence revealed a 648-kb inversion around the origin of replication. However, Southern blotting, PCR amplification and sequencing analyses of the inverted region revealed that the genome of *M. ap *K-10 differs from the published sequence in the region starting from 4,197,080 bp to 11,150 bp, spanning the origin of replication. Additionally, two new copies of the coding sequences > 99.8% were identified, identical to the MAP0849c and MAP0850c genes located immediately downstream of the MAP3758c gene.

**Conclusion:**

The optical map of *M. ap *ATCC 19698 clearly indicated the miss-assembly of the sequenced genome of *M. ap *K-10. Moreover, it identified 2 new genes in *M. ap *K-10 genome. This analysis strongly advocates for the utility of physical mapping protocols to complement genome sequencing projects.

## Background

*Mycobacterium avium *subspecies *paratuberculosis *(*M. ap*) is the causative agent of Johne's disease. The complete genome sequence of *M. ap *K-10 was published in 2005 [1] revealing a single circular chromosome of 4,830 kb and 4,350 predicted open reading frames (ORFs). Roughly, 1.5% of the genomic DNA is repetitive sequences, many of which are IS elements including 17 copies of IS*900 *and seven copies of IS*1311 *[1]. Previously, comparative genomic hybridizations were utilized to examine the extent of genomic diversity among members of the *M. avium *complex including *M. avium *subsp. *avium *(*M. av*), *M. avium *subsp. *hominissuis *(*M. ah*) and *M. ap *using DNA microarrays [2,3]. In these studies, areas of genomic rearrangements (e.g. insertions/deletions, inversions) were found between *M. ap *and *M. av*, a reflection of the plasticity of mycobacterial genomes. However, no appreciable differences were found when the genomes of the sequenced strain, *M. ap *K-10 (a recent isolate from clinically infected cow) and the type strain, *M. ap *ATCC 19698 (a laboratory strain) were compared. Using DNA microarrays, gene order information (synteny) related to each of the genomes was not obtained because of the nature of DNA microarray analysis. Here, we applied optical mapping to examine the difference between those two strains, on a genome-wide scale.

Optical mapping is unique among methods for analyzing genomes in that large-scale organizational information about the genome is preserved by physical attachment of large DNA fragments to a surface and assembly of a restriction digestion map based on imaging of a large number of individual restriction-digested genomic DNA molecules bound to the surface [4,5]. Such physical maps have uncovered unique genomic elements and provided scaffolds for genome sequencing and validation efforts that include: *Deinococcus radiodurans *[6], *Rhodospirillum rubrum *[7], *Yersinia pestis *[8], *Plasmodium falciparum *[9] and two *Xenorhabdus *species [10], as well as comparative genomics of *Shigella flexneri*, *Yersinia pestis*, and *Escherichia coli *[11]. Comparative genomic analyses using optical mapping data readily discover and characterize gene duplications, indels and genomic rearrangements. In unique ways, the system accurately identifies genomic copy-neutral variations such as inversions and translocations, which compensates for analysis shortcomings of other genomic approaches such as comparative genomic hybridizations, restriction fragment length polymorphism and pulsed-field gel electrophoresis [12].

The main goal of this study was to examine variations between two closely related genomes by optical mapping, which had never been applied to mycobacteria. The complete genome sequence for one of the examined strains (*M. ap *K-10) is already available [1] while the genome of *M. ap *ATCC 19698, the type strain of the species, has not been sequenced. An optical map with a resolution of ~600 bp did not reveal significant indels between the genomes. However, the map indicated that a 648-kb region was inverted relative to the published genome sequence of *M. ap *K-10. Sequencing analysis revealed that the inverted region is flanked by repetitive sequences. Additionally we find that the MAP0008c gene is 45-bp longer and there are two additional ORFs nearly identical to IS*1311 *and IS_MAP03 that differ from the published sequence.

## Results

### The optical map of *M. ap *ATCC 19698

**Figure 1:**
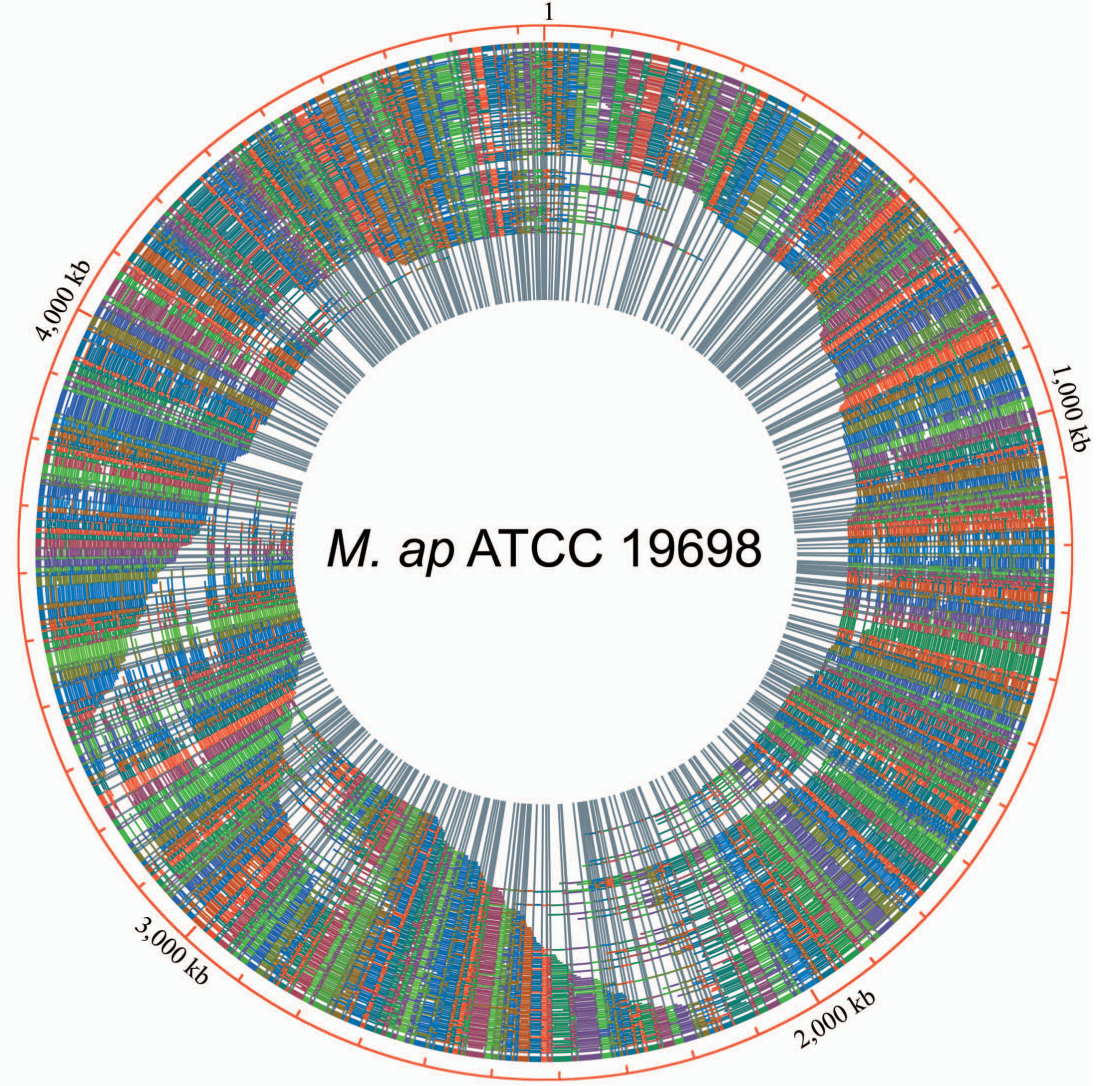
**A high-resolution optical map of *M. ap *ATCC 19698**. Genomic DNA of *M. ap *ATCC 19698 was mounted on derivatized glass using a microfluidic device and digested with *Bsi*WI. Fragment images were collected and processed by an automatic data acquisition system. A total of 970 optical contigs were assembled into one circular consensus map, giving an approximately 82-fold genome coverage. Optical contigs are represented by arcs of various lengths. Each arc is intersected by radiating lines that represent *Bsi*WI cutting sites, and arbitrary colors represent homologous overlapping fragments.

To generate an optical map of *M. ap *ATCC 19698, genomic DNA of the strain was digested with *Bsi*WI, and information of length and physical arrangement of the digested fragments were visualized and collected under a fluorescent microscope. To start the *de novo *assembly process of the *M. ap *ATCC 19698 optical map, we selected the largest ~5% of the optical contigs (larger than 550 kb in length) with average restriction fragment sizes less than 12 kb, and assembled these contigs to form a whole-genome map, termed an "optical consensus map". The predicted average size of *in silico M. ap *K-10 *Bsi*WI fragments was 7.3 kb, which guided our choice for average fragment size of less than 12 kb for *M. ap *ATCC 19698 genome assembly. A total of 970 high molecular weight gDNA (genomic DNA) contigs were acquired allowing for the construction of a genome-wide consensus map of *M. ap *ATCC 19698 with a summed mass of 396.102 Mb, or approximately 82-fold coverage of the *M. ap *K-10 genome (Figure 1). The *Bsi*WI final consensus map comprises 547 restriction fragments, which was 76 fragments less than those of the *in silico *map of *M. ap *K-10, mostly because fragments smaller than 600 bp or two adjacent fragments that are both smaller than 1 kb cannot be efficiently detected by optical mapping.

Additionally, three optical fragments (> 2 kb) were not aligned to the K-10 *in silico *map, most likely due to absence of *Bsi*WI sites on the ATCC 19698 DNA or because artificial *Bsi*WI sites on K-10 that were introduced by sequence errors. Based on the size of flanking regions, those fragments are not likely to be novel insertion sequences. The size of *M. ap *ATCC 19698 optical map was scaled according to its known G+C content for correcting raw fluorescence intensities measurements altered by very high G+C content [9]. Corrected estimates of the physical map revealed that the ATCC 19698 genome size is 4,839 kb, 9 kb > the sequence of *M. ap *K-10 (4,830 kb) [1]. This 9-kb difference includes additional sequences (2.8 kb) that will be described later in this article. The rest 6.2 kb represents 0.1% of the sequenced genome and within the expected error level when sizing fragments with fluorescent intensity.

### Alignment of the *M. ap *ATCC 19698 and K-10 restriction maps

**Figure 2:**
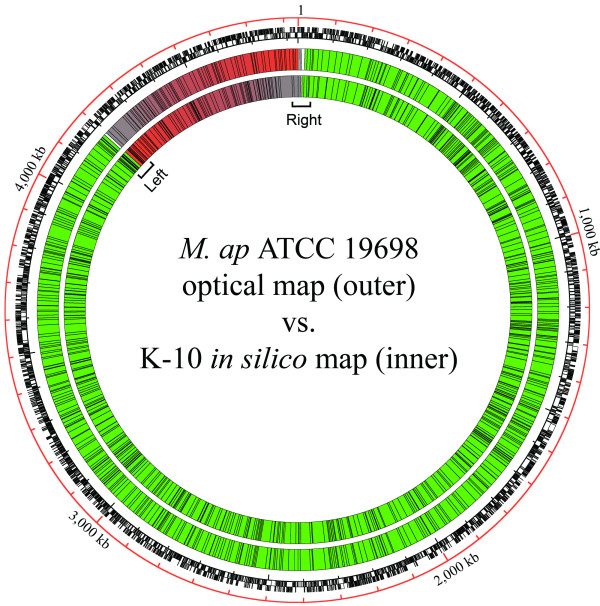
**Alignment of the *M. ap *ATCC 19698 and K-10 restriction maps**. Designations for the four concentric circles are as follows, from the outermost to innermost: genome coordinates, K-10 ORFs, ATCC 19698 *Bsi*WI optical map and K-10 *Bsi*WI *in silico *restriction map. Green boxes represent restriction fragments that are aligned between the genomes. The inversion region is shown in red-to-gray color gradient boxes. Possible inversion breakpoint regions are also indicated (Left and Right). The first ORF, MAP0001, is within the inversion segment, close to the right breakpoint.

To examine regions of difference between the *M. ap *strains, the assembled optical consensus map of *M. ap *ATCC 19698 was aligned to an *in silico *restriction map of *M. ap *K-10 with the *Bsi*WI restriction enzyme. Alignment of the genomes revealed a high degree of similarity between the genomes, given the ~600-bp resolution power [11] used in this study. However, the alignment revealed a ~648-kb segment (13.4% of the genome) consisting of 99 *M. ap *K-10 *Bsi*WI fragments that were inverted compared to the *M. ap *ATCC 19698 optical consensus map. A circular map aligning the annotated *M. ap *K-10 and *M. ap *ATCC 19698 genomes was constructed to illustrate the location of the inverted region (Figure 2), which interestingly, spans the origin of replication.

### Southern blotting analysis of the inverted region

**Figure 3:**
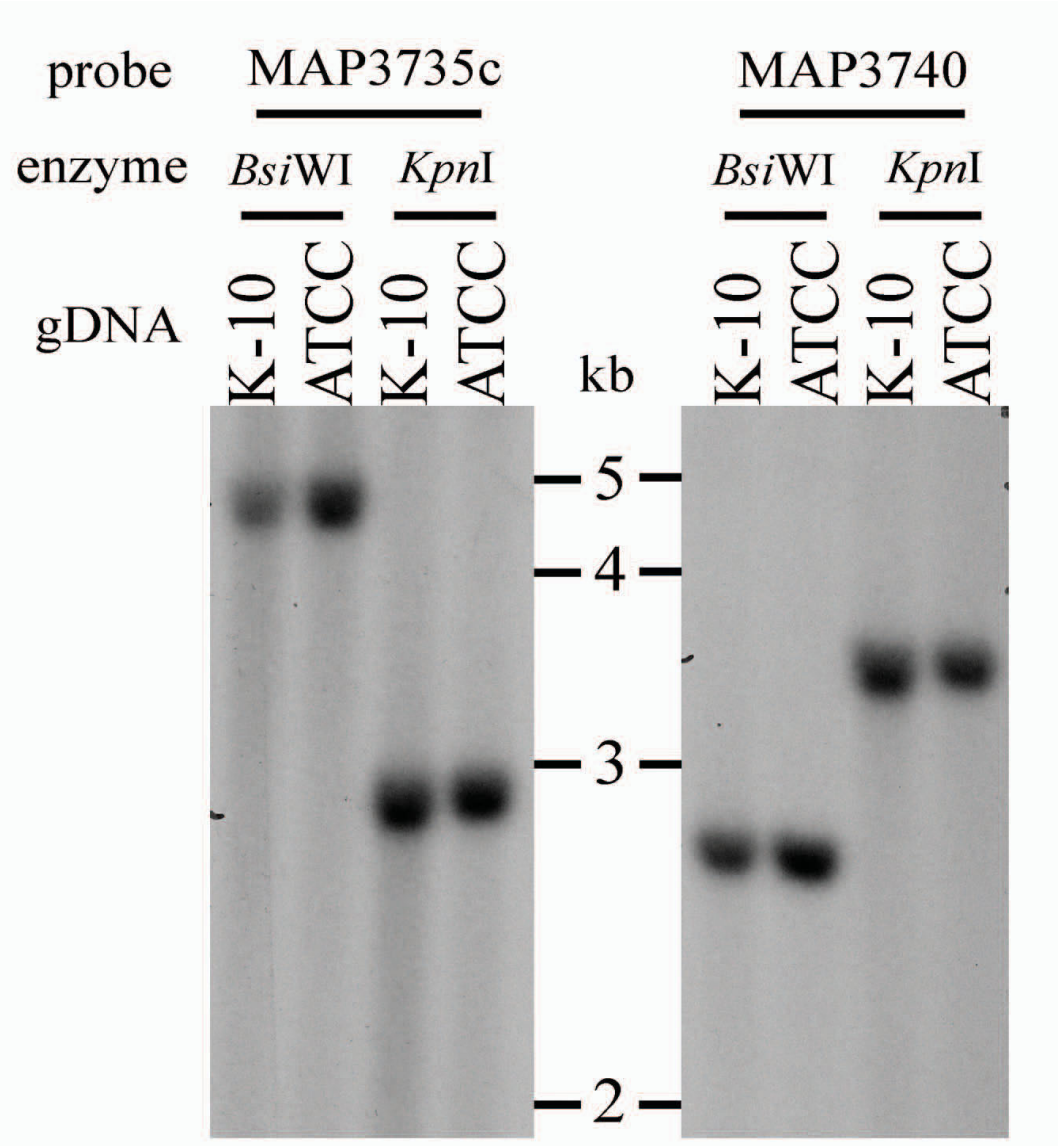
**Southern blotting analysis of the *M. ap *genomes**. Genomic DNA from the two strains were digested with either *Bsi*WI or *Kpn*I, and subsequently probed with labeled fragments indicated in blue boxes, in supplementary Figure Two in Additional file 1. Note the equal size of restriction fragments detected in both genomes with either probes.

To confirm possible breakpoints of the identified inversion fragment, we applied Southern blotting analysis to compare the fragment sizes from both genomes digested with *Bsi*WI and detected with ~850-bp probes (blue boxes in supplementary Figure Two, Additional file 1). The probes were designed either from an aligned fragment in the optical map (green boxes), or from a non-aligned fragment (white boxes) to validate the alignment pattern. In parallel, we also hybridized *Kpn*I-digested genomic DNA with the same sets of probes. Interestingly, the hybridization patterns showed no difference between the two genomes in the two examined fragments (Figure 3), in contrast to the result suggested by alignment of the published genome sequence to the optical map.

### PCR and sequencing analyses

**Figure 4:**
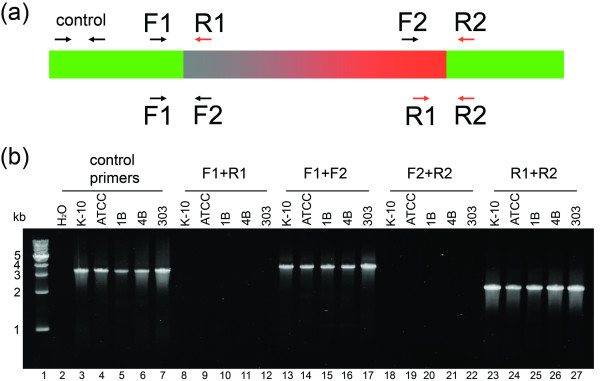
**PCR analysis of the inverted region in *M. ap *genomes**. (a) A diagram showing the inverted region and location of primers used in the PCR analysis. All primers were designed according to the published *M. ap *K-10 genome sequence. (b) An ethidium bromide stained agarose gel of the PCR reactions. Lanes of reactions amplified with original primer pairs F1+R1 (lane 8–12) or F2+R2 (lane 18–22) show no PCR products. Lanes of reactions amplified with switched primer pairs F1+F2 (lane 13–17) or R1+R2 (lane 23–27) show a 3.6-kb and 2.3-kb fragments, respectively. Each primer pair was used in reactions with genomic DNA from five *M. ap *laboratory strains or clinical isolates. ATCC, *M. ap *ATCC 19698; 1B and 4B, clinical isolates from humans; 303, a clinical isolate from a cow.

PCR primers were designed to amplify regions that contain possible inversion breakpoints. On the basis of the alignment between the two genomes, different patterns of amplification were expected from the genomes of *M. ap *K-10 and *M. ap *ATCC 19698. The average size of *Bsi*WI restriction fragments that were included in the map assembly was 10.8 kb, larger than the size of a regular PCR amplification. Therefore, we divided target DNA fragments into ~3-kb segments with several primer pairs to ensure efficient PCR amplification. When using a primer pair that would amplify a fragment containing an inversion breakpoint, an amplicon was expected from one genome but not from the other genome. We attempted to amplify a total of 24 fragments spanning the entire inverted region (see supplementary Table Two, Additional file 1, for details). Consistent with the Southern blotting results, we found that with all pairs of primers, the same sizes of products were obtained from both genomes (supplementary Figure One, Additional file 1). An example of the PCR analysis is presented in Figure 4. In this example, two pairs of primer combinations (Figure 4a, F1+R1 and F2+R2) were not able to amplify a 2.1-kb and 1-kb fragments (Figure 4b, lane 8, 9, 18, 19) that would be expected if the region was inverted between the genomes. Instead, when the primer pairs were switched (Figure 4a, F1+F2 and R1+R2), a 3.6-kb and 2.3-kb amplicons were amplified from both *M. ap *K-10 and ATCC 19698 (Figure 4b, lane 13, 14, 23, 24). This demonstrates that this region is not inverted in these genomes and that both strains share the orientation of this region suggested by the optical map of ATCC 19698. We cloned and sequenced these amplicons from both genomes and confirmed that sequences flanking the proposed inversion breakpoints are identical in both genomes.

Furthermore, to exclude possibilities that the optical mapping results were caused by genomic rearrangements during maintenance of the bacterial strains in our laboratory, we isolated gDNA from cultures of *M. ap *ATCC 19698 and K-10 obtained from another laboratory and subjected to PCR analysis. In addition, we analyzed the genomes of three other different clinical isolates (Figure 4b) from humans (1B and 4B) but with the IS*1311 *cattle genotype [13,14] and from a newly isolated strain from a cow (#303). Also, the 4B strain was analyzed before using DNA microarrays [2]. In all cases, the orientation of the inverted segment was agreed with the optical mapping results, indicating that this inversion was not recently introduced into *M. ap *ATCC 19698 in the laboratory.

### Alterations of the *M. ap *K-10 genome

**Figure 5:**
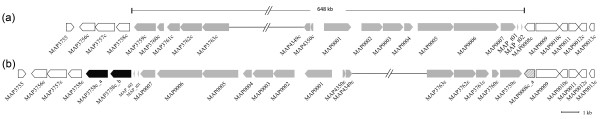
**Comparison of the previously-published (a) and revised (b) gene arrangements of the *M. ap *K-10 genome context examined in this study**. Arrows represent size, orientation and order of the ORFs. White arrows represent unchanged ORFs, Black arrows represent new ORFs, hatched arrow represents an altered ORF and gray arrows represent ORFs in the inverted segment.

On the basis of our analyses, we corrected the assembly of the genome sequence of *M. ap *K-10 starting from MAP3759c to MAP0007, corresponding to 4,197,080 bp to 11,150 bp in the genome of *M. ap *K-10 (Figure 5). This region includes the origin of replication and 606 predicted ORFs. According to our sequencing results, one of the two breakpoints is located between the fourth and fifth base pairs from the 3' end of MAP0008c (published coordinate 11,150). After we revised this region of the genome sequence, the MAP0008c ORF, now named MAP0008c_a, has a new stop codon located 45-bp downstream the breakpoint. Therefore, the MAP0008c protein is now 207 amino acids in length instead of 192 residues. Complete sequencing of the PCR fragments revealed two consecutively additional copies of ORFs, which are > 99.8% identical to insertion sequences IS*1311 *and IS_MAP03, located immediately downstream of MAP3758c (Figure 5). The two novel ORFs were named MAP3758c_a and MAP3758c_b. As a result, the segment is flanked by two copies of IS*1311 *(MAP3758c and MAP3759, Figure 5b). Supplementary Table Two in Additional file 1 details the order of genes in the inverted region. All new sequences (MAP0008c_a, MAP3758c_a and MAP3758c_b) are deposited in the GenBank database (accession numbers EU910154, EU910155 and EU910156, respectively).

## Discussion

Diversity of the genomic contents among strains of the same species of pathogens plays an important role in their evolution and could increase the antigenic repertoire of organisms to overcome host immune defenses. Genomic rearrangements including insertions/deletions or inversions are usually responsible for genomic diversity that is well-documented in members of *M. avium *complex including *M. ap *[13]. With the availability of complete genome sequences, pathogen diversity is usually analyzed on a whole-genome level. Unfortunately, high throughput sequencing projects are subject to errors and needs continuous improvement as technology progresses. One of the most encountered sequencing errors is single nucleotide miscalls, resulting in frameshifts or additions/deletions of ORFs [15-17]. Other errors such as inversions and translocations are usually associated with the assembly step of the whole sequence. In this study, we analyzed errors in the *M. ap *K-10 genome that are associated with sequence assembly.

Earlier microarray analysis of strains belonging to the *M. avium *complex identified large regions of insertions/deletions [18] in addition to 3 regions of large inversions between *M. av *and *M. ap *genomes [2]. Here, we used optical mapping [11] to examine genomic rearrangements between *M. ap *K-10, the recently sequenced strain, and ATCC 19698, the type strain of *M. ap*. Physical mapping has been conducted to compare genomes between *M. tuberculosis *and *M. bovis *[19] and between *M. tuberculosis *and *M. leprae *[20]. By taking the advantages of current technology, we are able to generate a high-resolution physical map of *M. ap*. Earlier, optical mapping was used for large-scale, comparative analysis of several genomes of enteric pathogens revealing loci responsible for serotype conversion[11]. Based on the comparison of genomic maps of *M. ap *ATCC 19698 and *M. ap *K-10, both genome sequences shared significant identity on a genome-wide scale at the resolution of the current optical mapping system [11]. In fact, combined estimated size of the *M. ap *ATCC 19698 genomes is only 6.8 kb (about 0.1%) larger than the size of the sequenced *M. ap *K-10 genome.

Surprisingly, comparing the generated optical map to the restriction map of the *M. ap *K-10 genome revealed an inversion of a large DNA segment (648 kb). The location of this inversion was close to an inverted region (inversion fragment III, 863.8 kb) that was identified earlier when *M. ap *and *M. ah *genomes were compared [2]. Southern blotting, PCR and sequencing analyses did not confirm the difference between these genomes. This suggests two possibilities. One possibility is that there is an error in the assembly of the published *M. ap *K-10 genome sequence and that it should be corrected to reflect the data in this report. A second possibility is that the changes reflect real mutational differences that have occurred during propagation of the *M. ap *ATCC 19698 in the laboratory. Notably, the K-10 strain in our lab was obtained from Dr. Raul Barletta, the same origin for the K-10 strain used in the genome project (personal communication). Accordingly, we performed PCR analysis on strains K-10 and ATCC 19698 maintained in another laboratory as well as clinical isolates from different sources (human and cow). This analysis confirmed the optical mapping data, resulting in a revised segment that is flanked by IS*1311*, suggesting an assembly error is the reason for the inversion.

Previously, optical mapping was used to help the assembly of the *Y. pestis *genome [8], a strategy that was not applied for *M. ap *K-10 sequencing project [1]. Interestingly, the MAP0001 gene and the origin of replication are included in the inverted region, which is usually used to dictate the orientation of genes in the genome. However, the *OriC *region and conserved sequences involved in replication (e.g. DnaA boxes) [21] remained intact in the revised sequence, therefore, the inversion of this region should not interfere with DNA replication. We suggest maintaining the same gene identification numbers in the inverted region to avoid confusions caused by changes. However, the genome sequence web portal should contain all of the information gathered from optical mapping (see supplementary Table Two, Additional file 1). Annotators and investigators interested in genomic synteny should be aware of the inversion in the assembled genome of *M. ap *K-10 strain. Alternatively, we could reassign the complete locus tags with a distinct prefix to reflect the revised gene order and orientation. However, this task will require the re-naming of the whole genome of *M. ap *K-10. Further inspection of the correctly assembled genome identified two additional copies of genes that were paralogues to known genes in *M. ap *K-10 genome, suggesting a gene duplication or transposition event. The importance of such gene duplication events remains to be analyzed in the future.

## Conclusion

In general, optical mapping used here validated the genome sequence of *M. ap *K-10 and showed its overall identity to the un-sequenced genome of *M. ap *ATCC 19698. Additionally, this study revealed differences from the published *M. ap *K-10 genome that should be further investigated. Specifically, the revised MAP0008c is 45 bp longer than the previously published one, and the additional sequence does not match to any known homologous domain, thus impact on the function of MAP0008c remains unknown. Despite the lower resolution level of optical mapping (~600 bp) compared to whole-genome sequence analysis (1 bp), optical mapping provides a less expensive approach to studying genomic rearrangements. We strongly support the notion of using physical mapping-based protocols to complement projects of whole-genome sequencing.

## Methods

### Bacterial strains

Laboratory strains of *M. ap *ATCC 19698 and K-10 [2] were the main strains used throughout this study. *M. ap *K-10 was obtained from Dr. Raul Barletta, University of Nebraska-Lincoln, and ATCC 19698 was from Dr. Mike Collins, University of Wisconsin-Madison during 2002 and 2003. Both strains have been kept at -80°C. The experiments were carried out from cultures growing from the frozen stock without further passages. The same strains were obtained from Dr. Mike Collins, during the verification stage of the optical mapping results in 2008. Other laboratory strains [2] isolated from humans (strains 1B and 4B, from Dr. Saleh Naser, Florida) and a cow (strain 303, from Dr. Mike Collins, Wisconsin) were used for PCR analysis. All strains were grown in Middlebrook 7H9 broth supplemented with ADC (10%) and Mycobactin J (2 gm/L). The IS*1311 *type of *M. ap *K-10, ATCC 19698, human 1B and human 4B are of cattle type [13,14], and the SSR type of the four strains are 15G/5GGT, 10G, 7G/5GGT and 7G/4GGT, respectively [22-24]. No genotyping analysis has been done on the cow strain 303.

### Genomic DNA preparation

To obtain high molecular weight genomic DNA (gDNA) with limited mechanical shearing, we applied a DNA extraction method for pulse-field gel electrophoresis [25]. A 500 μl *M. ap *ATCC 19698 culture at OD_600 _= 2.0 was pelleted, washed once and resuspended in equal volume of TE buffer. The bacterial suspension was mixed with 500 μl of 1% low melting temperature agarose at 45°C. The mixture was added to a 100-μl long rod-shaped mold to form an agarose insert. After 30 min, the agarose insert was transferred to a 15-ml capped tube with 5 ml bacterial lysis buffer (10 mM Tris-Cl pH7.6, 100 mM EDTA, 0.5% N-lauroyl sarcosine, 1 mg/ml lysozyme and 20 μg/ml RNase) and incubated at 37°C overnight. The buffer was replaced with 5 ml secondary bacterial lysis buffer (0.25 mM EDTA, 1% N-lauroyl sarcosine and 1 mg/ml proteinase K) at 55°C for 48 hrs with a change of buffer after 24 hrs of incubation. The agarose inserts were melted by incubation at 65°C for 6 minutes and digested at 42°C for 2 hours in a β-agarase solution (2 units of β-agarase in 100 μl TE). Lambda phage DNA (NEB, Ipswich, MA) was added to the sample at 25 pg/μl to serve as an internal sizing standard.

### Optical mapping

A standard protocol for optical mapping was adopted here as previously described [5,11,26] with a few modifications. Samples of isolated genomic DNA in solution were mounted on cleaned, derivatized glass surfaces using a microfluidic device [27] followed by polymerization of a thin layer of polyacrylamide (3.3% containing 0.02% Triton X-100). Surfaces were washed with 500 μl TE an 200 μl digestion buffer (NEBuffer 3 containing 0.02% Triton X-100) then digested for 3 to 4 hours with 20 units of *Bsi*WI (NEB). The surfaces were then rinsed twice with 500 μl TE and stained with 14 μl of 0.2 μM YOYO-1 (Molecular Probes, Eugene, OR). Fluorochrome-stained DNA fragments were imaged by fluorescence microscopy with a 63X objective lens (Carl Zeiss, Thornwood, NY) and a high-resolution digital camera (Princeton Instruments, Trenton, NJ). Images were collected using a fully automated image acquisition system, ChannelCollect [27] and processed with custom written Pathfinder software, which translates single DNA molecules into ordered restriction maps [7]. The sizes for restriction fragments were determined and assembled by the Pathfinder algorithm [6,28]. All of the remaining optical maps in the data set were then re-aligned to the consensus map, and re-assembled in an iterative fashion. Due to a difference in the G+C content of the *M. ap *genome versus intact lambda phage DNA and that YOYO-1 preferably intercalates GC pairs with higher quantum efficiency [29], a factor was used to correct the apparent size of fragments. OpGen Map Viewer (OpGen Technologies, Inc., Madison, WI) was used to align restriction maps generated from *M. ap *ATCC 19698 to the *in silico *restriction maps of *M. ap *K-10.

### Southern blot analysis

Samples of genomic DNA (gDNA) extracted from *M. ap *strains were analyzed by Southern blotting using a standard protocol that we developed earlier for *M. marinum *[30]. Briefly, 2 μg of gDNA was digested with either *Bsi*WI or *Kpn*I restriction enzyme (NEB) overnight and was electrophoresed on a 1% agarose gel and transferred to a nylon membrane (Perkin Elmer, Waltham, MA), using an alkaline transfer protocol as recommended by the manufacturer. A purified ~850-bp DNA fragment amplified with PCR (supplementary Table One, Additional file 1) was radiolabeled with [α-32P] dCTP using a random prime labeling kit (Promega, Madison, WI). Radiolabeled probes were hybridized to the nylon membrane at 65°C for 12 to 16 h in a shaking water bath before the membrane was washed and exposed to X-ray film (Kodak, Rochester, NY) before chemical processing to visualize hybridization signals.

### PCR and sequence confirmation

PCR primers used in this study are listed in supplementary Table One in Additional file 1. PCR reactions were performed in 25-μl reaction containing 1 M betaine, 50 mM potassium glutamate, 10 mM Tris-HCl pH 8.8, 0.1% Triton X-100, 2 mM magnesium chloride, 0.2 mM dNTPs, 0.5 μM each primer, 0.5 U of Taq DNA polymerase (Promega) and 25 ng of genomic DNA. The amplification thermocycle included an initial step of 94°C for 5 minutes followed by 5 cycles of 94°C for 30 s, 62°C for 30 s with 1°C decrease for each cycle and 72°C for 3.5 min, and followed by 30 cycles of 94°C for 1 minute, 57°C for 30 s and 72°C for 3.5 min. The single-band products from PCR reactions were subsequently cloned with the pGEM-T-easy cloning kit (Promega). Purified plasmid DNA was sequenced with BigDye Terminator v3.1 (Applied Biosystems, Foster City, CA) with primers listed in supplementary Table One in Additional file 1, according to the manufacturer's instruction. All sequences were analyzed with BLASTn algorithm on the NCBI web portal .

## Abbreviations

*M. ap*: *Mycobacterium avium *subspecies *paratuberculosis*; *M. av*: *Mycobacterium avium *subspecies *avium*; ORF: open reading frame; gDNA: genomic DNA.

## Authors' contributions

CW maintained bacterial stocks and cultures, performed genomic DNA extraction, Southern blotting, PCR and sequencing analyses and drafted the manuscript. TMS carried out optical mapping data acquisition. TMS and SZ performed optical map assembly and analysis. CW, DCS and AMT participated in designing the experiments. AMT coordinated and conceived of the study.

## Supplementary Material

Additional file 1 **Supplementary figures and tables.** Figures showing PCR and Southern blotting analysis of the inversion segment in *M. ap *K-10 and ATCC 19698, and tables listing the primers in this study and the new gene synteny identified by optical mapping and sequencing analysis. Click here for file
